# Barriers and facilitators to uptake and use of oral pre-exposure prophylaxis in pregnant and postpartum women: a qualitative meta-synthesis

**DOI:** 10.1186/s12889-024-19168-4

**Published:** 2024-06-20

**Authors:** Ying Liu, Liao Zhang, Hong Chen

**Affiliations:** West China Hospital, Sichuan University/West China School of Nursing, Sichuan University, Chengdu, Sichuan China

**Keywords:** HIV, Pre-exposure prophylaxis, Pregnancy, Barriers, Facilitators, Meta-synthesis

## Abstract

**Background:**

Acute HIV infection during pregnancy and in the postpartum period increases the risk of vertical transmission. The World Health Organization (WHO) has recommended preexposure prophylaxis for pregnant and postpartum women at risk of acquiring HIV. However, there are significant gaps between the actual practice and the ideal goal of preexposure prophylaxis implementation among pregnant and postpartum women. Therefore, it is important to determine what influences women’s implementation of preexposure prophylaxis during pregnancy and in the postpartum period. This review aims to aggregate barriers and facilitators to preexposure prophylaxis implementation among pregnant and postpartum women.

**Methods:**

A range of electronic databases, including PubMed, CINAHL Plus with Full Text, Embase, and Web of Science, were searched for potentially relevant qualitative studies. The search period extended from the establishment of the databases to March 16, 2023. This review used the ENTREQ (Enhancing transparency in reporting of qualitative research synthesis) statement to guide the design and reporting of qualitative synthesis. The methodological quality of the included studies was assessed using the Joanna Briggs Institute Critical Appraisal Checklist. The JBI meta-aggregation method was applied for guiding the data extraction, and the JBI ConQual method was applied for guiding the evaluation of the level of evidence for the synthesis.

**Results:**

Of retrieved 2042 studies, 12 met the inclusion criteria. The total population sample included 447 participants, including 231 pregnant and postpartum women, 21 male partners, 75 healthcare providers (HCPs)/healthcare workers (HCWs), 18 policymakers, 37 mothers, and 65 women of childbearing age. A total of 149 findings with credibility ratings of “unequivocal” or “equivocal” were included in this meta-synthesis. Barriers and facilitators to preexposure prophylaxis implementation were coded into seven categories, including three facilitator categories: perceived benefits, maintaining relationships with partners, and external support, and four barriers: medication-related barriers, stigma, barriers at the level of providers and facilities, and biases in risk perception.

**Conclusion:**

This systematic review and meta-synthesis aggregated the barriers and facilitators of preexposure prophylaxis implementation among pregnant and postpartum women. We aggregated several barriers to maternal preexposure prophylaxis implementation, including medication-related factors, stigma, barriers at the level of providers and facilities, and risk perception biases. Therefore, intervention measures for improving preexposure prophylaxis services can be developed based on these points.

**PROSPERO Number:**

CRD42023412631.

**Supplementary Information:**

The online version contains supplementary material available at 10.1186/s12889-024-19168-4.

## Introduction

There were still an estimated 1.3 million new infections in 2022, and HIV remains a major global health issue [1]. Of those newly infected, approximately 42% are adolescent girls and women (age ≥ 15 years) [1]. Evidence has shown that pregnant and postpartum women are at up to 2 to 4 times higher risk of acquiring HIV than their nonpregnant and postpartum period [2]. Factors contributing to increased HIV susceptibility during pregnancy include increased innate and suppressed adaptive immunity, increased genital tract inflammation, alterations in vaginal microbiota, decreased vaginal epithelium integrity, and gross or microtrauma to the genital tract [2, 3]. Furthermore, acute HIV infection during pregnancy and the postpartum period is an important reason of vertical transmission of HIV [4, 5]. Among the causes of new HIV vertical transmission reported in 2020, acute maternal or breastfeeding infection accounted for 23% of the new vertical transmission [6]. Therefore, prevention of maternal HIV infection is crucial to eliminate vertical transmission and reduce the global HIV prevalence.

To prevent the HIV epidemic, a series of HIV biomedical interventions have been developed, including a so-called ‘test and treat’ combination, treatment as prevention, vaccination and oral pre-exposure prophylaxis (PrEP) [7]. PrEP is a highly effective biological prevention method for individuals at high risk of HIV [8]. The global adoption of PrEP has been on the rise, with over 90 countries approving it for HIV prevention by December 2022 [9]. The World Health Organization (WHO) has also recommended that PrEP is commenced for any individual at risk of HIV acquisition, including the use of PrEP for pregnant and postpartum women at risk of exposure to HIV [10]. Previous studies indicate that oral PrEP use before, during, and after pregnancy does not pose an increased risk of adverse pregnancy outcomes [11–13]. Despite these recommendations and several maternal health benefits, both knowledge and uptake of PrEP among pregnant and postpartum women still remain low [14]. PrEP counseling and services for cis-gender women, including those who are pregnant or postpartum, currently remain limited [15]. Even in regions with high HIV incidence, such as Africa, PrEP programs have primarily focused on men who have sex with men, and the delivery of PrEP to women during pregnancy and postpartum is still in its early stages [16].

While previous studies have investigated the experience and influencing factors of maternal oral PrEP from a single perspective, these isolated perspectives cannot comprehensively summarize the key factors affecting the implementation of maternal oral PrEP [17, 18]. To date, researchers have not identified a published systematic review examining the factors influencing the implementation of maternal oral PrEP. Therefore, it is necessary to aggregate the barriers and contributing factors to the implementation of maternal oral PrEP from multiple perspectives. We then used a meta-aggregation approach to conduct a qualitative and systematic review of barriers and facilitators to oral PrEP in pregnant and postpartum women. Meta aggregation is grounded in the philosophic traditions of pragmatism and Husserlian transcendental phenomenology, and it is usually used to produce recommendations to guide practitioners and policymakers [19]. The purpose of this review is to aggregate the barriers and facilitators of maternal oral PrEP from multiple perspectives, and to provide intervention directions for future acceptance and implementation of oral PrEP for pregnant and postpartum women.

## Methods

### Design

The protocol for this review was pre-registered with PROSPERO (PROSPERO, CRD42023412631) and followed the PRISMA and ENTREQ (Enhancing transparency in reporting the synthesis of qualitative research) conduct and reporting guidelines [20, 21]. JBI’s meta-aggregation approach was used to guide the data extraction and synthesis, and the JBI ConQual approach was used to evaluate the evidence level of the synthesized findings [22]. This meta-synthesis of qualitative studies was conducted to aggregate barriers to and facilitators of oral PrEP among pregnant and postpartum women and to answer the following two questions: What are the barriers to oral PrEP use among pregnant and postpartum women? What factors can facilitate oral PrEP among pregnant and postpartum women?

### Search strategy

We conducted searches across four databases: PubMed, CINAHL Plus with Full Text, Embase, and Web of Science. The search period extended from the establishment of the databases to March 16, 2023. The search strategies were the combination of medical subject headings (MeSH), title, abstract, keywords and Boolean operators (AND/OR/NOT). Key search terms were grouped into themes relating to HIV, oral PrEP, and qualitative study. To avoid omitting potentially relevant studies, we did not limit the participants or countries of the articles during the search process. The details of all search strategies we used are available in Supplementary Material 1.

### Eligibility criteria

The primary studies were selected following the PICoS format (participants, phenomenon of interest, context, and study design).

Studies were included if they met the following criteria:

1. Participants: All studies with an aim to qualitatively identify and report barriers and facilitators to oral PrEP in pregnant and postpartum women were eligible;
2. Phenomenon of interest: Potential barriers and facilitators to oral PrEP use among pregnant and postpartum women;
3. Context: We did not restrict background conditions for this review;
4. Study design: Qualitative research with no limitation of the methodology (i.e., phenomenology, ethnography or grounded theory method), and mixed-method studies were included if they offered clear qualitative analysis and the primary data could be extracted.

### Exclusion criteria

The exclusion criteria included the following:

1. Review articles, conference abstracts, posters, books, and dissertations;
2. Studies that did not identify or discuss oral PrEP among pregnant and postpartum women;
3. Repeated publications;
4. Studies did not have available full texts, and.
5. Studies were not an English article.

### Study selection

**Fig. 1:**
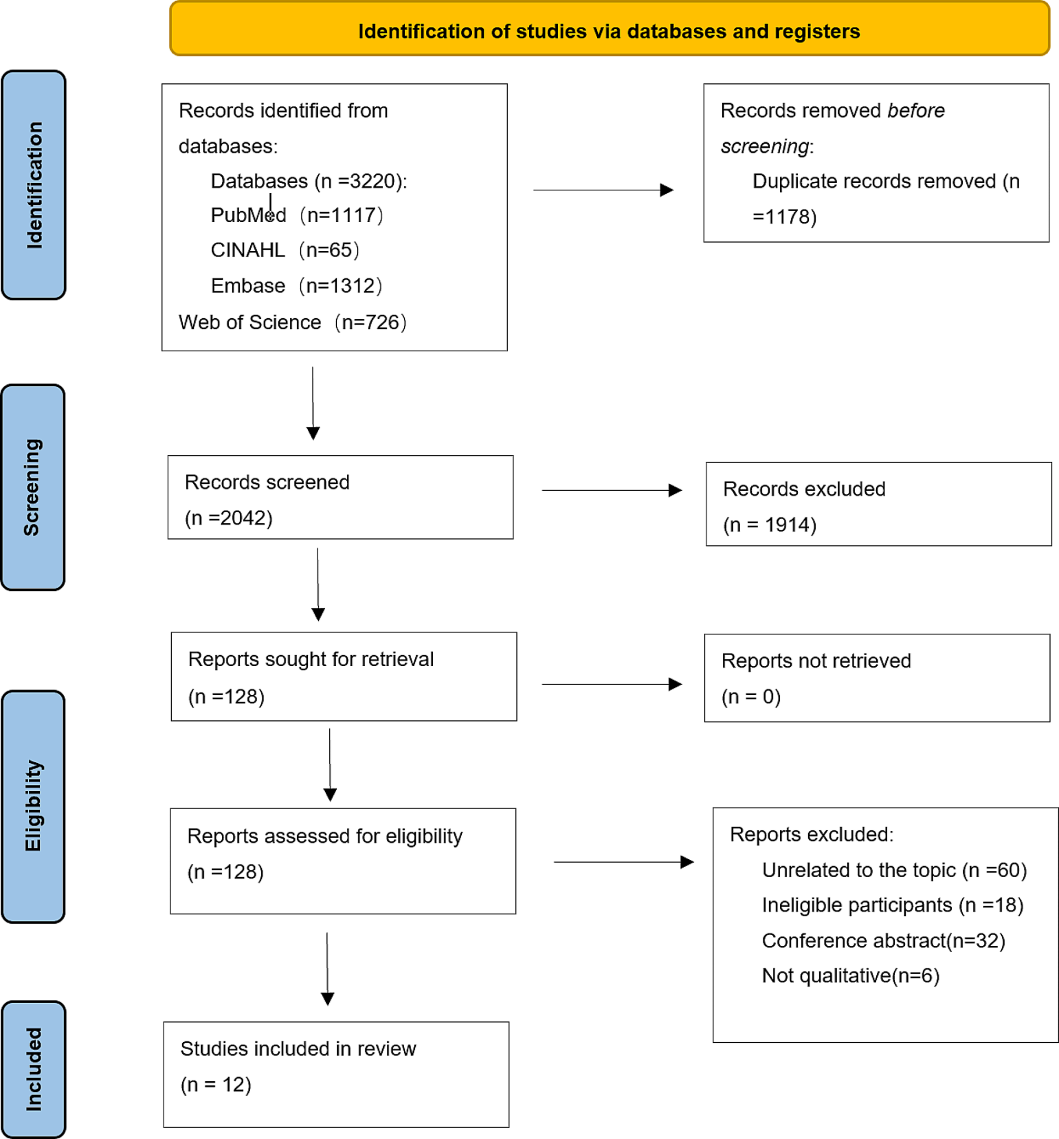
Flow diagram of search strategy and study selection

All retrieved articles were imported into the reference management program Endnote X9 and duplicates were removed. Two authors (Liu and Zhang) independently undertook the screening process, following the PRISMA guidelines (see Fig. 1). Two researchers (Liu and Zhang) independently screened the titles and abstracts of the studies following our inclusion criteria. Afterwards, the full text of potentially relevant studies was read to select eligible articles for inclusion in this review and meta-synthesis, and the reasons for excluded studies were classified in detail. Any disagreement in the selection process was discussed among two researchers or consultation with a third researcher (Chen) until agreement was reached.

### Quality appraisal

The methodological quality of included studies was investigated via the Joanna Briggs Institute Critical Appraisal Checklist for critical and interpretive research [23]. This checklist consists of 10 items, each with four scoring criteria: “yes”, “no”, unclear or “not applicable”. If 60% of the items answered “yes”, the quality of the study was considered acceptable, 70–90% answered “yes” referred to good quality, and if 100% of the items answered “yes”, the quality of the study was high. A study was included if the item achieved a minimum of 60% “yes”. Two reviewers (Liu and Zhang) independently conducted the critical appraisal of each research synthesis selected. Moreover, discussions during a team meeting were held to resolve any disagreements.

### Data extraction

Data extraction occurred in two phases. The first phase of data extraction was the extraction of general details of the study, which was conducted by the same two researchers using a pre-designed Excel spreadsheet. The following study characteristics and outcomes were extracted (1) basic study information (first author, publication year, country, research setting); (2) study design (research objectives, sample size, methods, sampling methods, data collection and analysis methods); (3) outcome measures. The second phase of data extraction is the extraction of findings. Findings were defined as verbatim extracts of the author’s analytical interpretation of the results or data. When extracting research results, levels of “credibility” should be assigned based on the reviewer’s assessment of the degree of fit or agreement between the data and the accompanying exemplar quotes. There are three levels of “credibility”. A finding was rated as “unequivocal” if the congruence of the finding and the illustration accompanied was beyond a reasonable doubt, as “equivocal” if a clear association between them was lacking, or as “unsupported” if the data did not support the findings. Only unequivocal and equivocal findings were included, and unsupported findings were not presented in the synthesis result [23]. The extracted information was validated by a third investigator (Chen), and any disagreements were discussed with a third researcher (Chen) until consensus was reached.

### Data synthesis

JBI’s meta-aggregation approach was used to guide the data synthesis. This approach is grounded in the philosophic traditions of pragmatism and Husserlian transcendental phenomenology. It is a widely used method with the pragmatic aim of systematically reviewing qualitative research to generate synthesized findings that can be used to inform healthcare practice or policy, which is perfectly aligned with the purpose of this review [23]. The data synthesis was conducted using a three-stage process. First, extracting findings from the included studies (this is the second phase of the data extraction as well). Second, pooling the findings into new categories based on the similarity in meaning, and each category consisted of at least two findings. Third, developing one or more synthesized statements of at least two categories.

### Quality appraisal of each synthesized finding

The JBI ConQual approach was used to evaluate the dependability and credibility of each synthesized finding [24]. Dependability was assessed using five items (items 2, 3, 4, 6, and 7) from the JBI critical appraisal checklist. Dependability was rated high if 4–5 items were appraised with “yes”, moderate if 2 to 3 items were appraised with “yes”, and low if 0–1 item was appraised with “yes”. Credibility is a rating of findings and illustrations (direct citations) in the studies. Credibility is evaluated as follows: unequivocal, equivocal, and unsupported. If most included studies in a synthesized finding had a dependability rating of high/moderate/low/, the dependability of the synthesized finding remained “high”/degraded 1 level/degraded 2 levels. The overall credibility of a synthesized finding remained “high” if it consisted of unequivocal findings, degraded 1 level if it consisted of a mixture of unequivocal and equivocal findings, degraded 2 levels if it consisted of equivocal findings, degraded 3 levels if it consisted of a mixture of equivocal and unsupported findings, and degraded 4 levels if it consisted of unsupported findings. The overall ConQual score was rated with “high”, “moderate”, “low”, and “very low”, started with “high” and was downgraded one level for every downgrade in the dependability and credibility scores.

## Result

### Search results

The defined search strategy identified 3220 citations, of which 1178 articles were removed due to duplication, while 2042 potentially relevant studies were retained for further screening. Screening of titles and abstracts of remaining articles for their eligibility resulted in exclusion of 1914 obviously irrelevant records. The full texts of the remaining 128 studies were assessed for eligibility, leading to the exclusion of 116 studies that did not meet the inclusion criteria. Ultimately, 12 studies were critically appraised and included in the review. A flow diagram of the study selection process is provided in Fig. 1.

### Study characteristics

A total of 12 papers were included in the review [16–18, 25–33]. Of these 12 papers, four were described as descriptive qualitative studies, one was a mixed method study with thematic analysis of qualitative results, and the remaining seven qualitative research articles did not explicitly report the research methods. Among the 12 included studies, 11 studies conducted in Africa, with South Africa and Kenya being the most common; only one study addressed the perceptions and acceptance of oral PrEP among African American women. A total of 447 participants were enrolled in the studies, including 231 pregnant and postpartum women, 21 male partners, 75 healthcare providers (HCPs)/healthcare workers (HCWs), 18 policymakers, 37 mothers, and 65 women of childbearing age. The details of the included studies are shown in Table 1.

**Table 1:** Study characteristics

Author year	country	participant	setting	Aim	Sample size	method of data collection and analysis	Methodologic& sampling approach	Major Theme
Jillian PINTYE, MPH, 2017	Kenya	21 pregnant women	A private room at the clinic	The perceptions, motivations, and beliefs of HIV-uninfected women about PrEP use during pregnancy	21	Semi-structured interview Constant comparison method	Qualitative Descriptive Research Purposive sampling	1.Maintain HIV-serodiscordant partnerships 2. Keeping women and infants HIV-free 3. Had concerns over PrEP side effects and safety 4. Health providers have a positive influence on adherence to PrEP
Jillian Pintye, PhD,2018	Kenya	68 pregnant and postpartum women	A private room at the clinic	Perceptions of PrEP during pregnancy	68	Semi-structured FGD; content analysis	Qualitative Descriptive Research Purposive sampling	1.PrEP provides protection from straying partners 2.Confusion over using HIV treatment drugs for prevention 3.Strategies for avoiding potential social harm associated with PrEP use
Chifundo Zimba, 2019	Malawi and Zambia	39 HIV-negative pregnant/breastfeeding women, 14 male partners, 19 HCWs, and 18 policymakers.	Private rooms	PrEP acceptability and feasibility in antenatal and postpartum populations	90	IDIs; Inductive and deductive approaches	Qualitative descriptive approach convenience sampling	1.Knowledge about PrEP. 2.Opinions and perceived acceptability of PrEP 3 Individual -level implementation barriers and possible solutions. 4.Facility-level implementation barriers and possible solutions. 5.Policy-level implementation barriers and possible solutions.
Pia Juul Bjertrup, 2021	Eswatini	24 AGYW or PBW, and 11HCW	Private place	Structural and social factors that influenced PrEP use among young women and pregnant or breastfeeding women	35	IDIs and FGDs; Thematic analysis	Qualitative design Purposive sampling	1.PrEP as an enactment of agency and self-care 2. “PrEP for life” and pill fatigue 3.Social relations and their interactions with women’s agency to use PrEP
Dvora L. Joseph Davey,2021	South Africa	25 postpartum women	Private room/ Phone call	Facilitators of long-term maternal adherence	25	Semi-structured interview; thematic approach	Qualitative design Purposive sampling	Individual Factors; HIV‑Related Factors: Facility‑Level Factors:
Allison K.2022	U.S.	20 pregnant women.	Video conference	Perspectives on and preferences for PrEP for pregnant individuals	20	IDIs; content analytic approach	Qualitative design Purposive sampling	1 The perceived risk of HIV infection was low; 2 Little knowledge of PrEP; 3 Concerned about side effects of PrEP for unborn child; 4 Very few of their ob-gyns discussed PrEP as an HIV prevention tool with them. 5Participants’ preferences for oral PrEP formulations as compared with long-acting injectable PrEP formulations varied based on individual characteristics.
Esther Cathyln Atukunda,2022	Uganda	37 women of child bearing age and 7 male partners were interviewed.	NR	Factors influencing periconception and pregnancy PrEP uptake and use	44	IDIs; Content and dyadic analyses	Qualitative design Purposive sampling	1.Participant characteristics 2Individual level 3.Couple level 4.Community level
Ivana Beesham,2022	South Africa	21 postpartum women	Private room/ Phone call	Barriers to PrEP continuation and/or adherence.	21	IDI; thematic approach	Qualitative design Purposive sampling	1.Participant characteristics 2.Individual‑Level factors 3.Disclosure‑Related factors 4.Pill‑Related Factors 5.Clinical Setting/Facility‑Related Factors
Lauren M. Hill,2022	Malawi	30 pregnant women	Private room	Understanding women’s motivations and concerns for PrEP use	30	IDIs; thematic qualitative analysis	Mixed-methods study Purposive sampling	1.Factors motivating PrEP use 2.Concerns about PrEP use 3.Involvement and influence of others in PrEP decision-making 4.Feelings about PrEP decision
Shivali Joshi,2022	Uganda	11 mother and 12 women of child bearing age	NR	Attitudes, experiences and challenges with PrEP to understand what motivates or limits PrEP uptake and adherence during pregnancy	23	Semi-structured interview; framework analysis approach	Qualitative design Purposive sampling	1.Participants 2.Agency and trust 3.Safety 4.HIV and sex work-related stigma 5.Social support and societal perceptions 6.Access to PrEP and PrEP information
Nancy Mwongeli,2022	Kenya.	45HCWs, including 25 with experience providing PrEP and 20 without PrEP provision experience	Private room at the clinic	Understanding HCW beliefs about PrEP prior	45	Semi-structured intervie; content analysis	Qualitative design Purposive sampling	1.Oral PrEP is an Acceptable HIV Prevention Strategy and Meets the Needs of Pregnant and Postpartum Women 2.Knowledge Gaps Regarding Eligibility and Risk Assessment and HCW Attitudes Might Limit PrEP Implementation 3.Multiple Facility and Interpersonal Level Barriers may Limit the Feasibility of Oral PrEP Implementation
Monique A. Wyatt,2023	South Africa	4 pregnant women and 7 mothers and 14 women of child bearing age	Private space	Identify influences on PrEP adherence	25	Semi-structured interviews; matrix approach	Qualitative Descriptive Research Purposive sampling	1.Personal Characteristics of Qualitative Participant. 2.PrEP Adherence in the Qualitative Sample 3.Contextual Information on PrEP Adherence Influences

HCP = Health Care Provider PrEP = Pre-Exposure Prophylaxis AGYW = Adolescent Girls and Young Women PBW = pregnant and breastfeeding women IDI = in-depth interview HCW = Health Care Worker

NR = Not Report FGDs = focus group discussions

### Methodological quality

All studies had a clear statement of the research objectives and strong representation of the voices of participants in the studies, and had ethics approval from appropriate committees. Seven studies mentioned the location of the researcher culturally or theoretically [16–18, 25, 27, 30, 33]. Only five studies stated the influence of the researcher on the research [17, 18, 27, 30, 33]. The results of JBI-QARI assessment are shown in Table 2.

**Table 2:** Methodological quality

Study	Q1	Q2	Q3	Q4	Q5	Q6	Q7	Q8	Q9	Q10	Total percent of “Y”	Overall quality
Jillian PINTYE, MPH,2017	U	Y	Y	Y	Y	Y	Y	Y	Y	Y	90%	good
Jillian Pintye, PhD,2018	U	Y	Y	Y	Y	Y	Y	Y	Y	Y	90%	good
Chifundo Zimba,2019	U	Y	Y	Y	Y	Y	N	Y	Y	Y	80%	good
Pia Juul Bjertrup,2021	U	Y	Y	Y	Y	N	N	Y	Y	Y	70%	good
Dvora L. Joseph Davey,2021	U	Y	Y	Y	Y	Y	Y	Y	Y	Y	90%	good
Allison K.,2022	U	Y	Y	Y	Y	N	N	Y	Y	Y	70%	good
Esther Cathyln Atukunda,2022	U	Y	Y	Y	Y	N	N	Y	Y	Y	70%	good
Ivana Beesham,2022	U	Y	Y	Y	Y	Y	Y	Y	Y	Y	90%	good
Lauren M. Hill,2022	U	Y	Y	Y	Y	N	N	Y	Y	Y	70%	good
Shivali Joshi,2022	U	Y	Y	Y	Y	N	N	Y	Y	Y	70%	good
Nancy Mwongeli,2022	U	Y	Y	Y	Y	Y	Y	Y	Y	Y	90%	good
Monique A. Wyatt,2023	U	Y	Y	Y	Y	Y	N	Y	Y	Y	80%	good

(1) Is there congruity between the stated philosophical perspective and the research method? (2) Is there congruity between the research methodology and the research question or objectives? (3) Is there congruity between the research methodology and the methods used to collect data? (4) Is there congruity between the research methodology and the representation and analysis of data? (5) Is there congruity between the research methodology and the interpretation of results? (6) Is there a statement locating the researcher culturally or theoretically? (7) Is the influence of the researcher on the research, and vice- versa, addressed? (8) Are participants, and their voices, adequately represented? (9) Is the research ethical according to current criteria or, for recent studies, and is there evidence of ethical approval by an appropriate body? (10) Do the conclusions drawn in the research report flow from the analysis, or interpretation, of the data?

Note: Y = Y, N = No, U = Unclear

### Meta-aggregation

A total of 149 findings were extracted, and the barriers and facilitators to oral PrEP use were coded into seven categories, including three facilitators: perceived benefits, influence of partners with unknown serostatus, and external support, and the four barriers: medication-related barriers, stigma, barriers to the health care system, and biases in risk perception. The process of credibility evaluation and findings synthesis can be found in Supplementary Materials 2 and 3.

#### Synthesized finding 1: The facilitators of oral PrEP implementation in pregnant and postpartum women

We identified three categories of facilitators of oral PrEP implementation: perceived benefits of oral PrEP, maintaining relationships with partners, and external support.

#### Perceived benefits of oral PrEP

Pregnant and postpartum women perceive the protective effects of oral PrEP for both pregnant and postpartum women and newborns which is a strong motivation to use oral PrEP, especially in serodiscordant couples [16–18, 25–27, 29, 31]. The experience of taking oral PrEP during pregnancy and in the postpartum period and remaining HIV-free makes pregnant and postpartum women personally aware of the benefits of oral PrEP, which greatly facilitates the implementation of oral PrEP [34].

*“I did not want to give birth to a child who has HIV…And, even though we used condoms, That was also another reason that motivated me to continue using Truvada [during pregnancy], that in case of anything, Truvada was going to help me during the pregnancy.” (26-year-old woman)* [17].

Women can decide whether or not to initiate oral PrEP use, which gives them autonomy because there is no need to negotiate with their partner as with condom use. The use of oral PrEP reminds women of the importance of taking care of themselves and evoked their sense of responsibility and care for their life and self-worth [26, 32].

*Now when we have sex, we use condoms. Before I started PrEP, we weren’t using condoms. The pill (PrEP) encourages me to use condoms and to know that I will not end up getting infected (Woman 18–20 years)* [26].

#### Maintaining relationships with partners

Oral PrEP is a good method to protect against HIV acquisition, particularly for HIV-negative individuals in HIV serodiscordant partnerships, those with multiple sexual partners, and those who believed their partners had ancillary partners [25, 32]. When faced with HIV-serodiscordance, maternal initiation of oral PrEP is a way to maintain stability in their relationships and affirm their love and support for their HIV-Infected partner, which is an important facilitator of oral PrEP implementation [17, 29]. Choosing oral PrEP can provide a woman with protection against transmission and make her feel safe when deciding to stay in the relationship.

*“I was taking [PrEP] to motivate my husband to take ART. We set our medication time to be the same, so we take medicine together. I would take PrEP and he also takes ART and he would see that we are taking the drugs together.” (24-year-old woman)* [17].

#### External support

Healthcare providers play an important role in promoting oral PrEP adherence [20, 30–32, 36]. Their attitudes toward oral PrEP significantly influence pregnant and postpartum women’s initiation and continuation of oral PrEP [17, 18, 26, 27, 32]. Healthcare workers’ active support and attention to maternal oral PrEP will promote the adherence of pregnant and postpartum women to use oral PrEP throughout pregnancy and postpartum, which greatly promotes the implementation of maternal oral PrEP [17].

*“First the healthcare worker herself, the attitude of the healthcare worker, if I think it’s not a good idea, then it means I will talk less about it.”– PrEP-naïve nurse counsellor* [33].

Encouragement and support from family, friends, and male partners are also important facilitators of sustained maternal oral PrEP use, especially support and encouragement from male partners [25–27, 29, 33]. Male partners play a major role in the acceptability, use, and compliance of oral PrEP [33]. The positive attitude and support of partners greatly improve the acceptance and compliance of maternal PrEP, which is also an important promoting factor for maternal oral PrEP [35]. In general, external support perceived by pregnant and postpartum women is a great facilitator for maternal oral PrEP.

*“Like a woman who tells you she wants to take PrEP but wishes the husband would be there, she would have taken it.”– PrEP-experienced pharmacist* [33].

*“I would advise her to take [PrEP] whole-heartedly because the baby comes in contact with so many things in the womb, so you would find that you infect the baby. I would advise her to take it every day.”(Male partner, Zambia)* [25].

#### Synthesized finding 2: The barriers to oral PrEP implementation in pregnant and postpartum women

Four categories emerged regarding the barriers to oral PrEP implementation: medication-related barriers, stigma, barriers at the level of providers and facilities, and biases in risk perception.

#### Medication-related barriers

Safety and side effects of oral PrEP were reported as barriers in most of the qualitative studies [16, 17, 25, 27, 28, 30–32]. Pregnant and postpartum women may hesitate to take oral PrEP due to concerns about the safety of oral PrEP and the potential harm of side effects to the unborn baby and newborn. Women recognized that pregnancy symptoms and oral PrEP side effects were similar, making it challenging to distinguish between them and potentially leading to overreaction, which was seen as a potential barrier to continuing oral PrEP use during pregnancy [17].

*“The pregnant woman carries a baby in her womb. You have to ask yourself, maybe this baby of mine that is still in the womb can get miscarried or die [because of taking PrEP]. Also with the woman who is breastfeeding. Maybe this child she is carrying, if she eats the drug, it can affect the baby, so they will have thoughts or concerns [about using PrEP]” (20-year-old woman)* [17].

The adherence to daily pills was a big challenge of the oral PrEP implementation. Pregnant and postpartum women may frequently and intermittently forget to take their daily medication [29, 30, 33]. For example, a mobile lifestyle, such as leaving home, traveling, and moving, can make daily adherence to medication a challenge [16, 30]. And medication itself is one of the barriers to maternal oral PrEP adherence. Pregnant and postpartum women may refuse to start and continue oral PrEP because the tablets are too large, they do not like taking them, or they have to take other medications simultaneously [16, 30].

*“I was traveling mostly. I would sometimes leave it [PrEP] at [my flat] and go to [my home]. I would be at my other place for maybe 4 days without taking it.” PrEP user, lower adherer, age 30* [16].

*I don’t like pills, and I was also taking pills for the pregnancy. I thought these ones [PrEP] should wait because I was taking pills for the pregnancy…. I gave birth through a c-section [cesarean], so I stopped [PrEP]. I was taking pills for the operation, so I felt that they were too many. I put PrEP aside and focused on my baby and took the other pills, for the operation. (PID334, 29 years)* [30].

#### Stigma

A large proportion of qualitative studies have reported stigma as a barrier to maternal uptake of oral PrEP [16, 18, 27, 29–33]. Misconceptions about oral PrEP are prevalent in the community, with most people not understanding the difference between oral PrEP and ARVs and perceiving people taking oral PrEP as HIV positive. Consequently, pregnant and postpartum women may refuse oral PrEP due to fear of being perceived as HIV positive [29–32]. Additionally, using oral PrEP could indirectly reveal the couple’s serodiscordant status. Fear of being branded as “reckless and irresponsible” for choosing to stay with partners living with HIV led women to keep their use of oral PrEP secret, which became a barrier to its utilization in pregnant and postpartum women [29].

*“I cannot talk to anyone about it [PrEP] because people have different views about the drug. … yet in actual sense they have not bought the idea and even talk about you to other people telling them you are HIV positive” (under 20 years old, stable on PrEP, not pregnant).* [32]

*“The problem of sharing such things with others is that if I tell them, they will get to know that my husband has HIV… They will think I am reckless and irresponsible.” – Female, age 26, Low adherence (#102).* [29]

#### Barriers at the level of providers and facilities

Lack of health care resources was seen as a barrier to maternal oral PrEP implementation, which comprised of lack of human resources, lack of financial resources [25, 30, 32, 33]. For health care providers, maternal oral PrEP is complex and requires time spent counseling pregnant and postpartum women about the drug itself, risks, and adherence, increasing workload in an already overburdened clinical setting [33]. Not only that, but oral PrEP providers also described ambiguity in the definition of risk and lack of clarity on oral PrEP eligibility when prescribing oral PrEP, which is also a potential problem as a barrier to oral PrEP implementation [29, 32].

*… I haven’t reconnected with PrEP services because my baby and myself are no longer attending here in this clinic, I take my baby to a different facility for postnatal care and there hasn’t been any conversation about the PrEP service so I won’t know whether they offer it or not. (PID199, 28 years)* [30].

#### Biases in risk perception

The majority of studies reported pregnant and postpartum women had insufficient knowledge of sexual health related to HIV self-risk perception [16, 26, 29, 32, 33]. HIV self-risk perception refers to the individual’s perception of the possibility of being infected with HIV based on knowledge and behavior [36]. Pregnant and postpartum women who are clinically at high risk of HIV infection perceive themselves to be at low or no risk, but in reality, a lack of knowledge about their partner’s HIV status, coupled with suspicion of partner infidelity and most women not using condoms during sex, puts most women at high risk of HIV infection [33]. The discordance between self-perceived and actual risk may pose a challenge to the implementation of maternal oral PrEP.

*“The perception of the woman … Do they feel they are at risk? What kind of relationship do they have with their partner and especially about HIV because you see that perception is what will drive the appearance and what [will] drive their commitment to use of PrEP.”– PrEP-experienced community health worker* [33].

*“[W]hen you counsel somebody who is not sick, it’s difficult. Like*.

*you are telling me you want to prevent and but am not sick so I ask, ‘Why do you want to give me medication and am not sick?”– PrEP-experienced nurse counsellor* [33].

### Quality appraisal of synthesized findings

The quality appraisal of the synthesized findings is presented in Table 3. The overall ConQual score of all three synthesized findings was rated as “low”.

**Table 3:** Quality appraisal of synthesized findings

Synthesized finding	Dependability	Credibility	ConQual Score
The barriers of PrEP implementation	Down grade 1 level	Down grade 1 level	Low
The facilitators of PrEP implementation	Down grade 1 level	Down grade 1 level	Low

## Discussion

We conducted a qualitative meta-synthesis that included qualitative studies and a mix of studies reporting qualitative findings, aiming to uncover the barriers and facilitators that pregnant and postpartum women encounter when initiating and adhering to oral PrEP. Our findings indicate that several factors drive maternal oral PrEP use and adherence, including the perceived benefits of oral PrEP, maintaining relationships with partners and receiving external support. Notably, pregnant and postpartum women are highly motivated to initiate and consistently adhere to oral PrEP usage due to their desire to protect themselves from infection and give birth to an HIV-negative child. We also identified several challenges that participants encountered in initiating or consistently adhering to their oral PrEP regimen. These include concerns about drug safety and side effects, stigma surrounding oral PrEP use, provider-and-facility-level barriers, as well as biases in risk perception. Given the limited research on oral PrEP in pregnant and postpartum women, our findings may contribute to a deeper understanding of the barriers and facilitators to initiating and adhering to oral PrEP during pregnancy and in the postpartum period with high risk of infection, thereby providing support for future pregnant and postpartum women who receive oral PrEP.

Our findings indicated that perceived benefit was an important contributing factor to the implementation of maternal oral PrEP. Studies have shown that the benefits of protecting themselves and their babies from HIV infection are an attractive option for pregnant and postpartum women when explaining oral PrEP to women without knowledge or experience of oral PrEP [17]. In addition, multiple negative HIV testing results also make pregnant and postpartum women feel the benefits of oral PrEP and promote their adherence to oral PrEP [32]. Previous studies have shown that women are more motivated to address some health issues during pregnancy, and hence, motivation to take oral PrEP during pregnancy may be high, especially among high-risk women with unknown partner serologic status or HIV carriers [37–40]. To increase the perceived benefits of oral PrEP for pregnant and postpartum women, oral PrEP counseling should be offered to raise awareness of the advantages of oral PrEP for pregnant and postpartum women and increase their motivation to use this preventive measure [41].

Moreover, our finding of this systematic review was that external support had a large impact on promoting maternal oral PrEP implementation and adherence. A supportive environment, especially support from healthcare providers and partners, plays an important role in facilitating oral PrEP implementation [42].A positive and supportive attitude from these providers may significantly improve the implementation and compliance of PrEP for pregnant and postpartum women [17, 18]. At the same time, disclosing PrEP use to family and friends and obtaining support from them may improve maternal PrEP adherence. Moreover, in the context of a patriarchal society, if the partner expresses a positive and supportive attitude towards the implementation of maternal PrEP, the maternal attitude towards PrEP may be more positive and the compliance may be higher [35, 43, 44]. A supportive environment may inspire maternal confidence in oral PrEP [29]. This provides an important entry point to address maternal oral PrEP implementation and adherence, for example, encompassing the involvement of their parents or partners in oral PrEP promotion and health education initiatives, extending beyond solely targeting pregnant and postpartum women [45].

This review found that certain characteristics of oral PrEP pills may be barriers to the implementation of maternal oral PrEP. Foremost among these are concerns about the safety and side-effect profile of oral PrEP. Pregnant and postpartum women faced the challenge of distinguishing between normal pregnancy symptoms and oral PrEP-related side effects. They were concerned that the observed side effects might be a danger sign for oral PrEP-exposed infants, which greatly reduced the willingness to use oral PrEP [46, 47].Therefore, to alleviate maternal concerns about the side effects and safety of oral PrEP, medical providers should prioritize initiating early and frequent discussions with pregnant and postpartum women at high risk of HIV exposure, thoroughly explaining its safety profile, potential side effects, and providing informational support to enhance their awareness [48].

Furthermore, this review identified stigma against oral PrEP as a barrier to maternal oral PrEP implementation. Lack of knowledge about oral PrEP makes it difficult for the community to correctly distinguish between oral PrEP and antiviral therapy [49]. Pregnant and postpartum women may refuse oral PrEP for fear of being identified as HIV-positive individuals. Additionally, oral PrEP use may indirectly expose couples’ serodiscordant status, and the choice to stay with an HIV-infected partner for fear of being labeled “reckless and irresponsible” leads women to keep oral PrEP use secret, which may affect adherence [29]. According to the available literature, long-acting injectable PrEP has been found to potentially enhance adherence to HIV prevention measures among pregnant and postpartum women who face stigma associated with oral PrEP [50]. However, this approach should be complemented with additional strategies, encompassing community-based interventions that incorporate media and educational initiatives, as well as the active engagement of male partners in HIV prevention and education efforts [51, 52].

Our study found that a heavy healthcare burden is also a barrier to maternal prevention. For maternal oral PrEP services to be implemented, additional resources at the facility level are needed to ensure effective delivery of prevention, which include additional funding for provider training (clinical and operational), oral PrEP-specific information, education, and communication materials for clinic attendees, effective ways to promote adherence, and more. In addition, due to the lack of guidelines for screening women for oral PrEP, many healthcare providers have gaps in knowledge regarding eligibility review and risk assessment, and they are uncertain how to assess oral PrEP eligibility for pregnant and postpartum women [33]. To address this gap, it is imperative to provide healthcare providers administering oral PrEP with comprehensive training on its safety, efficacy, and proper prescription methods for pregnant and postpartum women [53]. Moreover, the integration of oral PrEP services into routine ANC clinics presents a chance to alleviate the current medical strain. A recent study underscores the potential of this integration to not only mitigate HIV incidence among pregnant and postpartum women, significantly decreasing perinatal transmission of HIV, but also to influence the overall HIV incidence rates [54].

At the same time, our study also found that there was a significant discrepancy between the perceived risk of self-HIV infection and the actual risk, which was a barrier for maternal oral PrEP implementation. In a Kenyan study among adolescent girls and young women (AGYW), 43% of those with at least one risk factor for HIV acquisition who did not initiate oral PrEP because they did not perceive themselves to be at risk [55]. Self-perceived risk and actual risk are not always congruent, pregnant and postpartum women often underestimate their risk of HIV infection, which seriously hampers the implementation of maternal oral PrEP. Future interventions should prioritize counseling services that effectively guide pregnant and postpartum women to conduct HIV risk self-assessments, thereby enhancing their risk perception regarding oral PrEP [56]. However, how to align maternal risk perception with their actual risk is not fully understood, and this may require more research [56].

### Limitation

Potential limitations to this qualitative systematic review relate to the challenges in identifying all relevant studies. First, the search was completed on 16th March 2023, and studies completed after this date were not included in this review. Second, we only included studies published in English, which may suggest that potentially relevant studies conducted in countries where English is not the predominant language may be missed. Third, although the included studies were all of good quality, almost all the included studies did not report the statement locating the researcher culturally or theoretically and the influence of the researcher on the research, which may weaken the overall quality of the evidence.

## Implications for future research and practice

To facilitate future oral PrEP implementation among pregnant and postpartum women and achieve the goal of eliminating mother-to-child transmission, future research and practice should:

- Carry out health education activities and popularize oral PrEP knowledge to improve awareness and address oral PrEP-related social stigma.
- Emphasize the important role of male partners in the implementation of maternal oral PrEP, we cannot ignore the positive impact of external support on pregnant and postpartum women and create a supportive environment for them.
- Increase human resources and financial investment and eliminate structural barriers to obtaining oral PrEP.
- Provide pregnant and postpartum women with choices of modalities for oral PrEP treatment.

## Conclusion

This review integrated the barriers and facilitators to maternal oral PrEP implementation during pregnancy and in the postpartum period following the JBI’s meta-aggregation approach. The studies we reviewed identified several common barriers to oral PrEP implementation including medication-related barriers, stigma, barriers at the level of providers and facilities, and biases in risk perception. Future initiatives to address barriers to maternal oral PrEP access could be based on reference to our findings.

### Electronic supplementary material

Below is the link to the electronic supplementary material.

Supplementary Material 1

## Data Availability

All data generated or analysed during this study are included in this published article [and its supplementary information files].
