# Supplementary material for: Barriers and facilitators to uptake and use of oral pre-exposure prophylaxis in pregnant and postpartum women: a qualitative meta-synthesis
Source: BMC Public Health. 2024 Jun 20;24:1653. doi: 10.1186/s12889-024-19168-4 (PMC11191323; doi:10.1186/s12889-024-19168-4)
Supplement: Supplementary file 1 — Supplementary Material 1 [file 12889_2024_19168_MOESM1_ESM.docx]

**Supplementary material 1:** The search strategies

| **Database** | **Search Strategies** |
| --- | --- |
| Pubmed | #1((((((((((HIV [MeSH Terms]) OR (HIV Infections [MeSH Terms])) OR (HIV[Title/Abstract])) OR (HIV/AIDS[Title/Abstract])) OR (human immunodeficiency virus [Title/Abstract])) OR (human immuno-deficiency virus [Title/Abstract])) OR (AIDS[Title/Abstract])) OR ( Acquired Immunodeficiency Syndrome [MeSH Terms]))) OR (acquired immuno-deficiency syndrome [Title/Abstract])) OR (acquired immune-deficiency syndrome [Title/Abstract])  Sort by: Publication Date |
|  | #2(((((Pre-Exposure Prophylaxis [MeSH Terms]) OR (PrEP[Title/Abstract])) OR (HIV PrEP[Title/Abstract])) OR (Pre-Exposure Prophylaxis[Title/Abstract])) OR (Preexposure prophylaxis[Title/Abstract])) OR (Pre exposure prophylaxis[Title/Abstract])  Sort by: Publication Date |
|  | #3((((((Qualitative Research [MeSH Terms]) OR (Qualitative Study [Title/Abstract])) OR (Qualitative Research [Title/Abstract])) OR (mixed method*[Title/Abstract])) OR (qualitative [Title/Abstract])) OR (interview*[Title/Abstract])) OR (phenomenology [Title/Abstract])  Sort by: Publication Date |
|  | #4 #1AND#2AND#3 |
| CINAHL | S1: TI HIV OR TI Human Immunodeficiency Virus OR TI HIV infection* OR TI Acquired Immune Deficiency Syndrome Virus  Expanders - Apply equivalent subjects Search modes - Boolean/Phrase |
|  | S2: TI PrEP OR TI Pre-Exposure Prophylaxis OR TI Pre exposure prophylaxis OR TI Preexposure prophylaxis  Expanders - Apply equivalent subjects Search modes - Boolean/Phrase |
|  | S3: TI Qualitative Study OR TI Qualitative Research OR TI mixed method* OR TI qualitative method* OR TI interview* OR TI experiences OR TI phenomenology OR TI ethnography OR TI grounded theory  Expanders - Apply equivalent subjects Search modes - Boolean/Phrase |
|  | S4: (TI Qualitative Study OR TI Qualitative Research OR TI mixed method* OR TI qualitative method* OR TI interview* OR TI experiences OR TI phenomenology OR TI ethnography OR TI grounded theory) AND (S1 AND S2 AND S3)  Expanders - Apply equivalent subjects Search modes Boolean/Phrase |
| Embase | #1 'human immunodeficiency virus'/exp  #2 hiv: ab, ti OR 'human immunodeficiency virus': ab, ti OR 'hiv infection*': ab, ti OR 'aids virus': ab, ti OR 'acquired immune deficiency syndrome virus': ab, ti  #3 #1 OR #3  #4 'pre-exposure prophylaxis'/exp  #5'pre-exposure prophylaxis': ab, ti OR prep:ab,ti OR 'preexposure prophylaxis':ab,ti OR 'pre exposure prophylaxis': ab, ti  #6 #4 OR #5  #7 'qualitative research'/exp  #8 'qualitative study': ab, ti OR 'qualitative research': ab, ti OR 'mixed method*': ab, ti OR interview*: ab, ti OR phenomenology: ab, ti OR ethnography: ab, ti OR 'grounded theory': ab, ti  #9 #7 OR #8  #10 #3AND#6AND#9 |
| Web of Science | #1 (((TI=(HIV)) OR TI= (Human Immunodeficiency Virus)) OR TI= (HIV infection*)) OR TI= (Acquired Immune Deficiency Syndrome Virus)  #2 (((TI=(PrEP)) OR TI= (Pre-Exposure Prophylaxis)) OR TI= (Preexposure prophylaxis)) OR TI= (Pre exposure prophylaxis)  #3 (((((((TS= (Qualitative Study)) OR TS= (Qualitative Research)) OR TS= (mixed method*)) OR TS=(interview*)) OR TS=(experience)) OR TS=(phenomenology)) OR TS=(ethnography)) OR TS= (grounded theory)  #4 #1AND#2AND#3 |

**Supplementary Material 2** List of findings and credibility appraisal from eligible studies (N = 149 findings)

| Study | Findings | Credibility |
| --- | --- | --- |
| Jillian PINTYE, MPH,  2017 | 1.Stable and supportive relationships with partners | Equivocal |
|  | 2. Supported HIV-infected partners in ART adherence | Unequivocal |
|  | 3. Ensure protection against HIV for the infant | Unequivocal |
|  | 4. Desire for their infants to remain HIV-free | Unequivocal |
|  | 5. PrEP was effective in preventing HIV | Unequivocal |
|  | 6. No side effects reinforced the use beliefs | Unequivocal |
|  | 7. Similarity between pregnancy symptoms and side effects of PrEP and one may exaggerate the other. | Unequivocal |
|  | 8. Hcps were identified as a source of valid information on side effects of PrEP | Unequivocal |
|  | 9 Having a healthy, HIV-free infant outweighed PrEP side effects | Unequivocal |
|  | 10. Worried that fetal exposure to PrEP could lead to pregnancy loss or harm newborn | Unequivocal |
|  | 11. Having a healthy infant after using PrEP absolved their safety concerns. | Unequivocal |
|  | 12. Positive, well-established supportive relationships with hcps facilitated PrEP adherence throughout pregnancy and beyond | Unequivocal |
| Jillian Pintye, phd  2018 | 13.During pregnancy, a woman's partner may be infected with AIDS | Unequivocal |
|  | 14. PrEP was strategy for mitigating risk brought upon pregnant women by their male partners’ behavior. | Unequivocal |
|  | 15 Protecting infants from contracting HIV also made PrEP an attractive option. | Equivocal |
|  | 16 Understanding the concept of PrEP would not be a personal barrier to PrEP use | Unequivocal |
|  | 17 PrEP may result problems within the relationship and family | Unequivocal |
|  | 18. Male partners find that their pregnant partner reacts negatively, even violence to PrEP use | Unequivocal |
|  | 19. Community-level stigma against women to avoid PrEP access | Unequivocal |
|  | 20‘‘Hidden’’ or ‘‘secret,’’ would prevent community stigma among users | Unequivocal |
|  | 21. Concealing PrEP from male partners would be challenging during pregnancy | Unequivocal |
|  | 22. Attend ANC as a couple so that clinicians could explain PrEP to male partners | Unequivocal |
| Chifundo Zimba  2019 | 23. Most perceived PrEP as a good method to protect against HIV acquisition | Equivocal |
|  | 24. Prevent HIV transmission to their unborn children | Unequivocal |
|  | 25. More information is needed before the final decision is made | Unequivocal |
|  | 26. Half of the partners supported use of PrEP during pregnancy or breastfeeding | Equivocal |
|  | 27Health care systems would manage PrEP because there are no guidelines to screen women for PrEP | Equivocal |
|  | 28Prioritize PrEP whether it would jeopardize current investments in HIV treatment | Unequivocal |
|  | 29. Concerns about the side effects of PrEP, including potential harm to the baby | Unequivocal |
|  | 30. Worried about the need for strict adherence | Unequivocal |
|  | 31. Additional resources at the facility level to ensure that PrEP would be delivered effectively | Unequivocal |
|  | 32. Long term funding and sustainability of PrEP, when relying heavily on donor agencies, | Unequivocal |
|  | 33.The need for monitoring and evaluation tools | Unequivocal |
| Pia Juul Bjertrup  2021 | 34. Protect themselves and/or their baby from HIV infection even overcome financial barriers | Unequivocal |
|  | 35. PrEP evoked responsibility and care for one’s own life and self-worth | Equivocal |
|  | 36. PrEP helps to achieve education and career | Unequivocal |
|  | 37. Pill fatigue and discontinued use | Unequivocal |
|  | 38. Partner serostatus was not known | Unequivocal |
|  | 39Struggled to assess clients’ current risks opting for a simpler and clearer message of the need for PrEP “for life”: | Unequivocal |
|  | 40. Nondisclosure and lack of shared decision making are barriers to PrEP | Unequivocal |
|  | 41. Take PrEP to bring negative reactions from partners | Unequivocal |
|  | 42PrEP use by their partners might provoke feelings of insecurity and lack of trust within a relationship |  |
|  | 43. The partner expressed understanding about taking PrEP | Unequivocal |
|  | 44 Young women without children disclose PrEP use to co-habiting family members could lead to negative comments and attitudes | Unequivocal |
|  | 45. Teenage children’s parents were reportedly opposed PrEP | Unequivocal |
| Dvora L. Joseph Davey,  2021 | 46. Motivation of HIV‑Free Infant | Unequivocal |
|  | 47Protecting the baby reinforced and influenced women’s thinking | Unequivocal |
|  | 48The HIV negative of the child is the effect of PrEP | Unequivocal |
|  | 49. The risk of HIV infection comes from partners | Unequivocal |
|  | 50. Pregnancy exacerbates fear of risky sexual behavior by a partner | Unequivocal |
|  | 51.Distrust of the serostatus of the partner | Unequivocal |
|  | 52.Women do not have a choice about protection | Unequivocal |
|  | 53 Encouragement makes to start or continue taking PrEP | Unequivocal |
|  | 54A few women expressed concern about anticipated conflict or discouragement but were pleasantly surprised by their partners’ responses. | Unequivocal |
|  | 55. More information is needed to receive PrEP | Unequivocal |
|  | 56. Partner, family and friend encouragement following disclosure about taking PrEP was a significant driver in PrEP adherence. | Unequivocal |
|  | 57 Concerns about disclosing PrEP use because of community stigmatization | Unequivocal |
| Allison K. | 58Concerns About PrEP Safety and Experiences of Side Effects | Unequivocal |
|  | 59 Participants learned about PrEP and appreciated that education was integrated into ANC | Unequivocal |
|  | 60. The counseling was also a key motivator for starting and staying on PrEP. | Unequivocal |
|  | 61. Recommended continuing the offer of PrEP integrated into antenatal care | Unequivocal |
|  | 62. Participants’ knowledge of PrEP was low | Equivocal |
|  | 63. Conversations about PrEP for HIV prevention were largely absent from antenatal encounters. | Unequivocal |
|  | 64Their ob-gyn is a credible source of information | Unequivocal |
|  | 65Conversations between obstetricians and pregnant women are informative | Unequivocal |
|  | 66Surprise to be able to consult an obstetrician for more information | Unequivocal |
|  | 67Probably be a good idea like for me to take it [PrEP] for any risk | Equivocal |
|  | 68The choice of PrEP is affected by many factors | Unequivocal |
|  | 69Forget to take PrEP every day | Unequivocal |
| Esther Cathyln Atukund’2022 | 70. The perceived benefits of PrEP helped some women overcome barriers | Unequivocal |
|  | 71Taking a daily pill when well, stigma, alcohol use | Unequivocal |
|  | 72 Motivations to have a healthy child encouraged high adherence | Unequivocal |
|  | 73 Serial HIV testing inspires PrEP confidence and motivated this woman’s adherence. | Unequivocal |
|  | 74Women who were unsure of their partner’s ART adherence behaviour and/or serostatus used PrEP | Unequivocal |
|  | 75Male partners with HIV appreciated knowing their partners had added protection | Unequivocal |
|  | 76Women with high adherence described using cues | Unequivocal |
|  | 77 Nondisclosure of PrEP use and HIV serostatus contributed to tensions, mistrust, gender stereotypes, suspicions of infidelity and gender-based violence. | Equivocal |
|  | 78Some women were suspected of infidelity when their partners discovered they were using PrEP and experienced violence | Equivocal |
|  | 79 Some women’s decision not to initiate PrEP, despite eagerness for HIV prevention benefits, was driven by anticipated violence | Unequivocal |
|  | 80For couples in mutually disclosed partnerships, PrEP was a tool in a “shared battle” against HIV | Unequivocal |
|  | 81 PrEP helped couples confidently meet their reproductive goals | Unequivocal |
|  | 82 Stigma related to HIV, antiretroviral drugs (ARVs) and serodifferent partnerships, and worries about unintended serostatus disclosure challenged PrEP adherence. | Unequivocal |
|  | 83Others using PrEP successfully inspired PrEP optimism and encouraged women to persist with PrEP | Unequivocal |
|  | 84.Keep PrEP use secret, which may impact adherence | Unequivocal |
|  | 85 PrEP efficacy perceptions and adherence were improved through ongoing support from study staff and health workers. | Unequivocal |
| Ivana Beesham,2022 | 86PrEP offered a strategy for one couple to support their reproductive goals and maintain their partnership | Unequivocal |
|  | 87 Forgetful to Take PrEP Daily | Unequivocal |
|  | 88 Being Away from Home When PrEP Needed to be Taken | Unequivocal |
|  | 89Feared that people would think that they were HIV-infected and taking ART. | Equivocal |
|  | 90 Stigma anticipated or experienced stigma influenced PrEP initiation, adherence and continuation | Unequivocal |
|  | 91Women reported multiple concerns regarding their partners’ response | Unequivocal |
|  | 92 Disclosure‑Related Factors ( disclosure was not reported as a barrier to PrEP use or continuation) | Unequivocal |
|  | 93Yet most women reported that such negative responses did not affect their PrEP continuation | Equivocal |
|  | 94 Anticipated or Experienced Side Effects | Unequivocal |
|  | 95Other Pill‑Related Factors eg: taking multiple medications (dislike for taking tablets, difficulties with committing to daily pill taking and challenges taking PrEP in addition to other tablets) | Unequivocal |
|  | 96Taking multiple medications allows for overlapping side effects | Unequivocal |
|  | 97Increased barriers to postpartum PrEP access which included transport, logistical, financial and access related barriers to consistent clinic attendance | Unequivocal |
| Lauren M. Hill,2022 | 98 Protecting themselves from HIV ;protect their unborn baby | Unequivocal |
|  | 99Motivation may have been particularly strong women in serodiscordant relationships. | Unequivocal |
|  | 100Some signalling mistrust at their partner’s avoidance of HIV testing | Unequivocal |
|  | 101 Suspected or anticipated partner non-monogamy as a central motivation for using PrEP. | Unequivocal |
|  | 102Some women discussed knowledge of current or past partner non-monogamy, which motivated their desire for HIV protection | Unequivocal |
|  | 103Concerned about the risk of birth defects, miscarriage and pre-term delivery | Unequivocal |
|  | 104Concerns regarding nausea, dizziness, weakness and physical appearance changes | Unequivocal |
|  | 105 Fears of being stigmatized as a result of PrEP use and other negative reactions from family and friends | Unequivocal |
|  | 106Confusion about HIV status and the difference between PrEP and ART | Unequivocal |
|  | 107Worried about their ability to adhere to PrEP | Unequivocal |
|  | 108Impact of partners on PrEP (they made the decision to use PrEP on their own) | Equivocal |
|  | 109 Very few women discussed PrEP with other family members before making their decision | Unequivocal |
|  | 110 Protection against HIV outweighed their concerns | Unequivocal |
|  | 111 It hard to say no to the HIV protection offered by PrEP | Unequivocal |
| Shivali Joshi,2022 | 112Desires for autonomy over their sexual health and livelihood | Unequivocal |
|  | 113Mistrust in partners influenced their desire to take PrEP | Unequivocal |
|  | 114Those who were unable to ensure condom use during sexual interactions were more motivated to take PrEP | Unequivocal |
|  | 115 PrEP allowed women choices within their occupation as well as opportunities for future occupations | Unequivocal |
|  | 116 Inability of society to keep YWHR and the sex worker community safe | Unequivocal |
|  | 117 The safety or lack of safety of PrEP was a large determinant as to whether participants sought out PrEP and continued to take the drug (PrEP-related side effects) | Unequivocal |
|  | 118 Safety of high-risk work while pregnant | Unequivocal |
|  | 119PrEP-related stigma (the high-risk community on PrEP is stigmatized similarly to individuals who are HIV positive.) | Unequivocal |
|  | 120 Unable to share that they were on PrEP with friends, family, and partners | Unequivocal |
|  | 121 Due to stigma, and thus not initiating or persisting taking PrEP because not sexually active | Unequivocal |
|  | 122 Stigma in health care Settings contributes to difficult access to PrEP | Unequivocal |
|  | 123 Information influences the decisions and choices of participants | Unequivocal |
|  | 124 Did not know of any organizations, individuals/communities, or systems for support for pregnant sex workers and high-risk women | Unequivocal |
|  | 125Did not know about the existence of PrEP. | Equivocal |
|  | 126 It is difficult for women to find PrEP in health facilities and physically access it | Unequivocal |
| Nancy Mwongeli,2022 | 127PrEP is safe in pregnancy and can avert new HIV infections in this population | Unequivocal |
|  | 128 PrEP could overcome gender disparities that hinder uptake of HIV prevention strategies, | Unequivocal |
|  | 129 PrEP gives women autonomy of choice | Unequivocal |
|  | 130 The demand and the need for PrEP in this population was high, and that pregnant and postpartum women would easily accept PrEP | Equivocal |
|  | 131 There could be misalignment between a woman’s perceived risk and her actual risk | Unequivocal |
|  | 132 PrEP implementation is highly dependent on the knowledge and attitudes that HCWs have towards PrEP. | Unequivocal |
|  | 133 Many HCWs were uncertain about how PrEP eligibility should be assessed among pregnant and postpartum women | Unequivocal |
|  | 134Health care workers' attitudes will influence women's PrEP | Unequivocal |
|  | 135 PrEP would increase the workload in already thinly stretched healthcare facilities | Unequivocal |
|  | 136 Taking a pill daily would be a huge burden for pregnant and postpartum women and they may experience challenges with PrEP adherence | Equivocal |
|  | 137 Women’s partners played a major role in the acceptability, use, and adherence to PrEP | Unequivocal |
|  | 138Community stigma could prevent PrEP uptake among women who want to use PrEP | Unequivocal |
| Monique A. Wyatt,2023 | 139 Proximity to the medication made regular dosing easier (Starts and stops appeared to map onto women’s changing proximity to partners) | Unequivocal |
|  | 140Mobile Lifestyle (The impact of mobility on PrEP adherence) | Unequivocal |
|  | 141Pregnancy and Motherhood（ worried about the potential consequences of combining PrEP with other medications they might need to take as pregnant women. ） | Unequivocal |
|  | 142Feeling destabilized made adhering to PrEP more difficult | Equivocal |
|  | 143Fear of PrEP-related Stigma（Fear of stigma made women reluctant to disclose their PrEP use） | Unequivocal |
|  | 144Disclosure of PrEP Use（Disclosure eliminated many barriers to adherence） | Unequivocal |
|  | 145Presenting PrEP as one of a number of safer conception strategies they were practicing tended to elicit a positive response from partners | Unequivocal |
|  | 146Perception HIV Risk（HIV risk to their partner） | Unequivocal |
|  | 147The risk of AIDS comes from partners’ presumed risky sexual behavior and/or unknown HIV status | Unequivocal |
|  | 148Protecting themselves against HIV infection | Unequivocal |
|  | 149 Side effects or a negative interaction with alcohol, and concerns about potential adverse effects on an unborn child | Unequivocal |

**Supplementary Material 3** Summary of study finding, categories, and synthesized categories to generate synthesized findings on the barriers, facilitators, and recommendations of PrEP implementation

| **The number of findings** | **Categories** | **Synthesized findings** |
| --- | --- | --- |
| 3，4，5，6，9，11，14，15，16，23，24，34，35，36，46，47，67，72，73，81，98，110，111，112，115，127，128，129，130，145，148 | Perceived benefits | The facilitators of PrEP implementation |
| 1,2，13，38，49，50,51，74，86，99，100，101，102，113，114，147 | Maintaining relationships with partners |  |
| 8，12，25，26，43，53，55，56，60，64，65，75，80，83，85，123，132，134 | External Support |  |
| 7，10，29，30，37，48，58，69，84，87，88，91，95，96，103，104，117，118，136，139，140，142，149 | Medication-related barriers | The barriers of PrEP implementation |
| 17,19,20,57,71,82,89,90,105,106,119,121,122,138,143 | Stigma |  |
| 27,28,31,32,33,61,63,97,116,124,125,126,133,135 | Barriers at the level of providers and facilities |  |
| 39,62,125,146,131 | Biases in risk perception |  |

**Supplementary material 4** ENTREQ checklist (Enhancing transparency in reporting the synthesis of qualitative research)

| **No. Item** | **Guide Questions/Description** | **Reported on Page** |
| --- | --- | --- |
| 1. Aim | This review aims to aggregate barriers and facilitators to preexposure prophylaxis implementation among pregnant and postpartum women. | P1 |
| 2.Synthesis methodology | Identify the synthesis methodology or theoretical framework which underpins the synthesis, and describe the rationale for the choice of methodology (e.g. meta-ethnography, thematic synthesis, critical interpretive synthesis, grounded theory synthesis, realist synthesis, meta-aggregation, meta-study, framework synthesis) | P5 |
| 3. Approach to searching | The search was pre-planned. Comprehensive search strategies were undertaken to seek all available studies. | P3-P4; Supplementary Material 1 |
| 4.Inclusion criteria | The primary studies were selected following the PICoS format (participants, phenomenon of interest, context, and study design). Studies were included if they met the following criteria: 1) Participants: according to our aim of this review, all studies with an aim to qualitatively identify and report barriers and facilitators to PrEP in pregnant women were eligible. 2) Phenomenon of interest: the (potential) barriers and facilitators to PrEP use among pregnant women. 3) Context: We did not restrict background conditions for this review. 4) Study design: Qualitative research with no limitation of the methodology (i.e., phenomenology, ethnography or grounded theory method), and mixed-method studies were included if they offered clear qualitative analysis and the primary date could be extracted.  The exclusion criteria included the following: 1) Review articles, conference abstracts, posters, books, and dissertations. 2) Studies that did not identify or discuss PrEP among pregnant women, 3) Repeated publications, 4) Studies that did not have available full texts, and 5) Studies was not an English article. | P4 |
| 5. Data sources | Four databases were searched: PubMed, CINAHL Plus with Full Text, Embase, and Web of Science. The search period extended from the establishment of the databases to March 16, 2023. The search strategies were the combination of medical subject headings (MeSH), title, abstract, keywords and Boolean operators (AND/OR/NOT). Key search terms were grouped into themes relating to HIV, PrEP, and qualitative study. To avoid omitting potentially relevant studies, we did not limit the participants or countries of the articles during the search process. The details of all search strategies we used are available in Supplementary Material 1 | P4-P5 |
| 6.Electronic Search strategy | Supplementary Material 1 describes the literature search | Supplementary Material 1 |
| 7.Study screening methods | All articles returned from the searches were imported into the reference management program Endnote X9 and duplicates were removed. Two authors (Liu and Zhang) independently undertook the screening process, following the PRISMA guidelines (see Fig. 1). Two researchers (Liu and Zhang) independently screened the titles and abstracts of the studies following our inclusion criteria. Afterwards, the full text of potentially relevant studies was read to select eligible articles for inclusion in this review and meta-synthesis, and the reasons for excluded studies were classified in detail. Any disagreement in the selection process were discussed among two researchers or consultation with a third researcher (Chen) until agreement was reached. | P4 |
| 8.Study characteristics | Table 1 presents the characteristics of the included studies (author(s), year of publication, country, setting, aim, sample size, methodologic & sampling approach, method of data collection and analysis, major theme | Table 1 |
| 9.Study selection results | A flow diagram using PRISMA guidelines for reporting of systematic reviews is presented in Figure 1 in reporting of the selection process and results. | Figure 1 |
| 10. Rationale for appraisal | The methodological quality of included studies was investigated via the Joanna Briggs Institute Critical Appraisal Checklist for critical and interpretive research^21^. There are 10 items in total, and each item has four scoring criteria, which are “yes”, “no”, unclear or “not applicable”. If 60% of the items answered "yes", the quality of the study was considered acceptable, 70-90% answered "yes" referred to good quality, and if 100% of the items answered "yes", the quality of the study was high. A study was included if the item achieved a minimum of 60% “yes”. Two reviewers (Liu and Zhang) independently conducted the critical appraisal of each research synthesis selected. Moreover, discussions during a team meeting were held to resolve any disagreements. | P4 |
| 11.Appraisal items | 2020 version of Checklist for Qualitative Research (Critical Appraisal tools for use in JBI Systematic Reviews) | P4; Table 2 |
| 1. Appraisal   process | Two reviewers (Liu and Zhang) independently conducted the critical appraisal of each research synthesis selected. Moreover, discussions during a team meeting were held to resolve any disagreements. | P4-P5 |
| 13.Appraisal results | Appraisal results are presented in Table 2 | Table 2 |
| 14.Data extraction | The first phase of data extraction was the extraction of general details of the study, which was conducted by the same two researchers using a pre-designed Excel spreadsheet. The following study characteristics and outcomes were extracted (1) basic study information (including the first author, publication year, country, research setting,); (2) study design (including research objectives, sample size, methods, sampling methods, data collection and analysis methods); (3) outcome measures. The second phase of data extraction is the extraction of findings. Findings were defined as verbatim extracts of the author's analytical interpretation of the results or data. When extracting research results, levels of “credibility” should be assigned based on the reviewer's assessment of the degree of fit or agreement between the data and the accompanying exemplar quotes. There are three levels of “credibility”. A finding was rated as “unequivocal” if the congruence of the finding and the illustration accompanied was beyond a reasonable doubt, as “equivocal” if a clear association between them was lacking, or as “unsupported” if the data did not support the findings. Only unequivocal and equivocal findings were included, and unsupported findings were not presented in the synthesis result^26^. The extracted information was validated by a third investigator (Chen), and any disagreements were discussed with a third researcher (Chen) until consensus was reached. | P5 |
| 15. Software | Endnote X9 was used to manage the citations. | P4 |
| 16. Number of reviewers | A minimum of two trained reviewers (Zhang, Liu, Song, and Chen) | PP4-5 |
| 17. Coding | JBI meta-aggregation did not use the technique of coding | NA* |
| 18.Study comparison | The process of aggregation involves the synthesis of findings by categorizing them through the similarity in meaning. Then, we subject these categories to a synthesis to generate more comprehensive findings. | P5 |
| 19.Derivation of themes | We subjected these categories to further synthesis to generate more comprehensive findings called synthesized findings. Only unequivocal and credible findings were included. Not supported findings were not presented in the synthesis or the results (Lockwood et al., 2015). | P5 |
| 20. Quotations | “No of finding file” provides findings and quotations from the primary studies to illustrate themes and constructs, and identify whether the quotations were participant quotations of the author’s interpretation. | Supplementary Material 2-3 |
| 21.Synthesis output | Synthesis output is presented in Supplementary material 2-3 | Supplementary Material 2-3 Summary of study findings, categories, and synthesized categories to generate synthesized findings on the barriers to and facilitators of HIV preexposure prophylaxis in pregnant and postpartum women |

^*^NA means 'not applicable'
